# Development, validation, and clinical evaluation of a machine-learning based model for diagnosing early infection after cardiovascular surgery (DEICS): a multi-center cohort study

**DOI:** 10.1097/JS9.0000000000002287

**Published:** 2025-02-04

**Authors:** Tuo Pan, Haitao Zhang, Chuangshi Wang, Hanghang Wang, Yusanjian Matniyaz, Zhi-Kang Lv, Tong Tong Zhu, Ya-Peng Wang, Zhi-Zhao Song, Yu-Xian Tang, He Zhang, Hao-Dong Pan, Chen Li, Lin-Shan Yang, Shi-Yu Guan, Wen Bian, Xiateke Hafu, Xiang Li, Yang Li, Xiao-Ting Wu, Zhi-Wei Fan, Yuan-Xi Luo, Yi Jiang, Ya-Xuan Gao, Wen-Zhe Wang, Yun-Xing Xue, Fu-Dong Fan, Jun Pan, Qing Zhou, Bo-Min Zhang, Wei Wang, Qiang Wang, Guo-Liang Fan, Dong-Jin Wang

**Affiliations:** a Department of Cardiac Surgery, Nanjing Drum Tower Hospital, Affiliated Hospital of Medical School, Nanjing University, Nanjing, China; b Department of Cardiac Surgery, Nanjing Drum Tower Hospital, Peking Union Medical College & Chinese Academy of Medical Sciences, Graduate School of Peking Union Medical College, Nanjing, China; c National Clinical Research Center for Cardiovascular Diseases, Fuwai Hospital, Chinese Academy of Medical Sciences & Peking Union Medical College, Beijing, China; d Division of Cardiac Surgery, Department of Surgery, Johns Hopkins Hospital, Baltimore, USA; e Heart Center, Massachusetts General Hospital, Harvard University, Boston, USA; f Department of Cardiac Surgery, Nanjing Drum Tower Hospital Clinical College of Nanjing University of Chinese Medicine, Nanjing, China; g Department of Clinical Medicine, Norman Bethune Health Science Center of Jilin University, Changchun, China; h Department of Critical Care Medicine, Shanghai East Hospital, School of Medicine, Tongji University, Shanghai, China; i Department of the Heart and Great Vessels, the affiliated Hospital of Yangzhou University, Yangzhou University, Yangzhou, China; j Yangzhou Institute of the Heart and Great Vessels (YIHV), Yangzhou University, Yangzhou, China; k Department of Cardio-Thoracic Surgery, Xinhua Hospital of Ili Kazak Autonomous Prefecture, Ili, China; l Department of Nosocomial Infection Control, Nanjing Drum Tower Hospital, The Affiliated Hospital of Nanjing University Medical School, Nanjing, China; m Department of Intensive Care Unit, The Affiliated Nanjing Drum Tower Hospital of Nanjing University Medical School, Nanjing, China

**Keywords:** antibiotics, cardiac surgery, early postoperative infection, machine learning, xGBoost

## Abstract

**Background:**

This study addresses the critical need for timely and accurate diagnosis of early postoperative infection (EPI) following cardiac surgery. EPI significantly impacts patient outcomes and healthcare costs, making its early detection vital.

**Objectives:**

To develop, validate, and clinically implement a machine-learning-based model for diagnosing EPI post-cardiac surgery, enhancing postoperative care.

**Methods:**

In this multi-center cohort study spanning 2020 to 2022, data from four medical centers involved 2001 participants. Of these, 1400 were used for trainingand 601 for validation. Several machines-learning algorithms, including XGBoost, random forest, support vector machines, least absolute shrinkage and selection operator, and single-layer neural networks, were applied to develop predictive models. These were compared against a traditional logistic regression model. The model with the highest area under the receiver operating characteristic curve (AUROC) was deemed optimal. Implemented across four centers since 1 January 2023, a retrospective real-world study assessed its clinical applicability. Among 400 patients with an estimated EPI risk above 10%, identified by the optimal model, 55 followed its antibiotic upgrade recommendations (DEICS group). The remaining 345 patients upgraded antibiotics empirically, with 55 in the control group, matched 1:1 with the DEICS group. Clinical utility was evaluated through antibiotic use density (AUD), hospital costs, and ICU stay duration.

**Results:**

The XGBoost model achieved the highest performance with an AUROC of 0.96 (95% CI: 0.93–0.98). The calibration curve exhibited strong agreement with Brier scores of 0.02. According to the XGBoost model, the DEICS group significantly demonstrated reduced AUD (*P* < 0.01) in the matched cohort, along with decreased ICU stay time (median: 5 vs. 6 days, *P* = 0.01) and hospital costs (median: ¥150 000 vs. median: ¥200 000, *P* = 0.01) in the EPI cohort.

**Conclusion:**

The successful implementation of the XGBoost model facilitates accurate EPI diagnosis, improves postoperative recovery, and lowers hospital costs.

## Introduction

Despite significant advancements in perioperative management of cardiac surgery, the incidence and mortality rates of postoperative infections have not substantially declined^[1]^. A recently published large-scale cohort study demonstrated that postoperative pneumonia is associated with a 7.4-fold increase in in-hospital mortality, while cohort studies from a decade ago found that postoperative infections increase mortality by 5- to 10-fold^[1–5]^. Our previous research indicated that 74.8% of postoperative infections occur within 48 hours of surgery, with pneumonia and sepsis being the predominant types^[6]^. These findings emphasize the critical importance of early detection and intervention for EPI to mitigate its impact on patient outcomes.

The accurate and timely diagnosis of EPI is fraught with challenges. Clinical symptoms of EPI often overlap with surgery-induced inflammatory responses, particularly after procedures involving extensive tissue trauma, cardiopulmonary bypass (CPB), or significant transfusion requirements^[6,7]^. While prophylactic antibiotics, primarily first- and second-generation cephalosporins, are standard practices, their efficacy against Gram-negative bacilli, a common EPI pathogen, remains suboptimal^[6,8,9]^.

The limitations of current diagnostic methods further exacerbate these challenges. The pathogen culture, the gold standard, is hampered by low sensitivity and a delay of 2–5 days for results, hindering timely and effective intervention^[6]^. This diagnostic gap often results in premature antibiotic escalation based on limited evidence, increasing the risk of resistance and complicating treatment^[6]^. However, studies have shown that extending prophylactic antibiotic use beyond 48 hours does not improve patient outcomes but may contribute to antimicrobial resistance^[6,10]^. Despite efforts to address this issue, existing predictive and diagnostic models lack the sensitivity and specificity needed for clinical implementation^[6,11–13]^.

The consequences of EPI extend beyond individual patients, placing significant strain on healthcare systems through increased resource utilization and costs. Addressing this issue is critical not only for improving patient outcomes but also for mitigating the broader public health challenge of antimicrobial resistance.

Emerging technologies such as artificial intelligence (AI) and machine learning (ML) offer a promising pathway for advancing the diagnosis and management of EPI^[14]^. These approaches have the potential to integrate diverse clinical and laboratory data, offering more accurate and timely diagnostic capabilities compared to traditional methods.

In this study, we aim to develop and validate a machine-learning based model specifically tailored for the diagnosis of EPI in cardiac surgery patients. By addressing the limitations of current diagnostic approaches, this model has the potential to facilitate the targeted use of antibiotics, reduce unnecessary escalation, and ultimately improve patient outcomes. The findings of this study could mark a significant step forward in the management of postoperative infections, addressing an urgent and unmet clinical need in cardiovascular surgery.

**Highlights**

- Developed a diagnostic model for early postoperative infections (EPI) based on a prospective cohort study.
- The XGBoost model achieved an AUROC of 0.96, outperforming traditional clinical judgment.
- Implementation led to significant reductions in antibiotic use, ICU stay duration, and hospital costs.

## Methods

### Study population

Phase 1: Model development. Conducted at NDTH from October 2020 to December 2021, this phase focused on prospectively collecting data aimed at developing various machine-learning models for diagnosing EPI.

Phase 2: Model validation. Spanning from January 2022 to December 2022, this phase entailed the external validation and comparison of the developed models. Data were prospectively gathered from four different medical centers. The models were assessed for accuracy, reliability, and applicability across diverse clinical settings, with the final model selected based on comprehensive comparisons of key performance metrics such as the area under the receiver operating characteristic curve (AUROC), sensitivity, specificity, and calibration. The chosen model demonstrated superior performance in accurately diagnosing EPI and adaptability across different hospital settings, thus being designated as the final model for clinical application.

Phase 3: Clinical utility of the model. Conducted from January 2023 to September 2023, this final phase evaluated the real-world efficacy of the selected model from Phase 2. Utilizing retrospective data from the four participating centers, this phase aimed to assess the model’s effectiveness in guiding postoperative care compared to traditional clinician judgment. Patients were matched for age, gender, and surgical procedure types to ensure validity. This phase provided critical insights into the practical utility of the model and its impact on patient outcomes across diverse healthcare environments.

This study was approved by the ethical committees of all participating centers, and written informed consent was obtained from all patients. The study’s reporting adhered to the Transparent Reporting of a Multivariable Prediction Model for Individual Prognosis or Diagnosis (TRIPOD) statement^[15]^ and Strengthening The Reporting Of Cohort Studies in Surgery (STROCSS) checklist^[16]^. The trial was registered at the Chinese Clinical Trial Register. The comprehensive study protocol has been published previously^[17]^. Patients or the public were not involved in the design, conduct, reporting, or dissemination plans of our research.

All three phases of the study used the same inclusion and exclusion criteria as follows: Patients aged between 18 and 80 years scheduled for cardiac surgery were considered for inclusion. Exclusion criteria consisted of the following:^[1]^ preoperative temperature ≥ 38°C^[2]^; surgery for trauma, infective endocarditis, tumors, and malignancies^[3]^; preoperative infections^[4]^; autoimmune disease or connective tissue disease^[5]^; pregnancy or breastfeeding^[6]^; and patients who died within 48 hours post-surgery.

Details on missing data imputation, the process of model development including sample size calculate, missing data imputation, feature selection, and outcomes evaluated are available in Supplemental Digital Content.

### Implementation across centers

The XGBoost model, identified as the most effective in Phase 2, was applied across the four medical centers, encompassing a total of 2400 patients. This model identified 400 patients as having an EPI risk above 10%, prompting recommendations for antibiotic treatment revisions. Of these 400 patients, 55 adhered to the model’s recommendation and had their antibiotic regimen upgraded at 7:00 am on the third postoperative day (POD3), forming the “DEICS group.” In contrast, 345 patients continued with existing empirical antibiotic strategies, which involved either continuation, extension, or modification of their regimen on POD3, based on clinician discretion. To assess the effectiveness of the model’s recommendations, a control group was formed from these 345 patients. This group was matched 1:1 with the DEICS group, accounting for age (±2 years), gender, and type of cardiac surgery. In the control group, the empirical criteria for early antibiotic upgrade included the presence of new or progressive radiographic infiltrate, fever above 38°C, leukocytosis (WBC > 12 × 10^9^/L) or leukopenia (WBC < 4 × 10^9^/L), and elevated inflammatory markers (IL-6 > 94 pg/ml, CRP > 68 mg/L)^[18]^. An upgrade of antibiotics was considered if at least two of these four criteria were met. Conversely, if none were met, the ongoing use of first- or second-generation cephalosporins was discontinued on POD3. Patients meeting only one of the four criteria were maintained on either first- or second-generation cephalosporin until normalization of CXR, WBC, CRP, or IL-6.

### Model development and internal validation

The first phase patients were used for model construction. Five machine-learning models – logistic regression with least absolute shrinkage and selection operator (LASSO), random forest (RF), extreme gradient boosting (XGBoost), support vector machine (SVM), and single-layer neural network (SLNN) – were developed and compared with logistic regression (LR) for diagnostic efficacy. Logistic regression models with LASSO regularization were constructed using the glmnet package in R, while RF, XGBoost, SVM, and SLNN models were built using the caret package. Specifically, the ‘rf’ method is selected for random forest modeling, and the ‘xgbtree’ method is chosen for XGBoost modeling. The parameter tuning process involves exploring different combinations of hyperparameter values, with a tune length of 3 iterations for RF and XGBoost models. The optimum tuning models were selected using 10-fold cross-validation, and model performance was assessed using calibration curves.

### External validation

External validation used patient data from NDTH (Nanjing Drum Tower Hospital), YZUH (Affiliated Hospital of Yangzhou University), SEH (Shanghai East Hospital), and ILXH (Xinhua Hospital of Ili Kazak Autonomous Prefecture). Model efficacy was measured using the area under the receiver operating characteristic curve (AUROC), sensitivity, specificity, positive predictive value (PPV), negative predictive value (NPV), accuracy, calibration curve, and Brier score.

### Statistical analysis

Analyses were conducted using R version 4.1.2. Categorical variables were summarized as frequencies (%) and compared using the chi-squared or Fisher’s exact tests. Continuous variables were presented as the median with interquartile range (IQR) and compared using the Mann–Whitney *U* test. To assess the balance of baseline variables, standardized mean differences (SMDs) were used, with SMD below 0.20 as indicative of acceptable balance. Statistical significance was set at *P* < 0.05, and all *P* values reported were two-sided.

## Results

**Figure 1.**
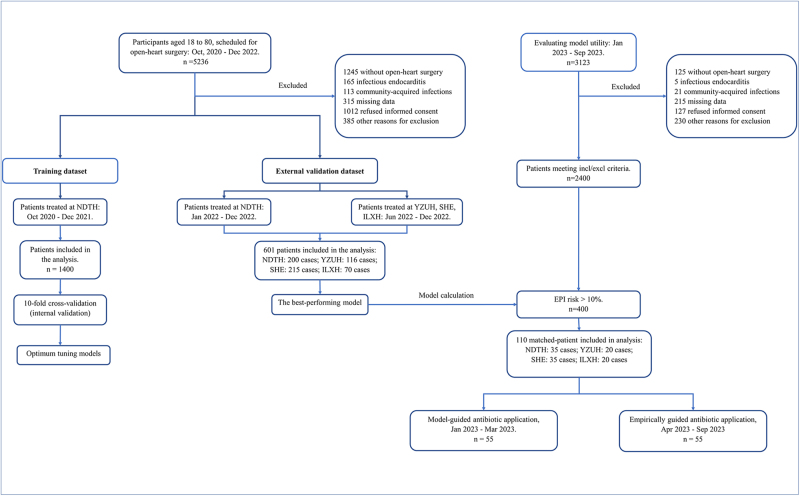
Flow chart.

During the study period from October 2020 to December 2022, we screened 5236 patients aged 18 to 80 years undergoing open-heart surgery. Of these, 3235 patients were excluded. The first phase at NDTH involved 1400 cases for model construction. The second phase included external validation with 200 cases from NDTH, 116 from YZUH, 215 from SEH, and 70 from ILXH. The third phase, for clinical application of the model, involved 35 cases from NDTH, 20 each from YZUH and ILXH, and 35 from SEH. The XGBoost-guided antibiotic treatment group (DEICS group) comprised 55 patients, and the control group, matched for age, gender, and surgical procedure, included another 55 patients (Fig. 1).

### Baseline characteristics

Table 1 presents a thorough examination of baseline features between the training and external validation datasets. Analysis of baseline characteristics revealed consistency across training and validation datasets, with no significant differences in age, gender, and BMI distributions. However, a significant difference was observed in the incidence of emergency surgery (*P* < 0.001), attributed to NDTH undertaking a higher proportion of emergency thoracic aortic surgeries (23.4% vs. 18.1%).

**Table 1:** Baseline features of training and validation datasets. Data are *n* (%) or median (IQR)

	Training dataset (*n* = 1400)	Validation dataset (n = 601)	*P*- value	SMD
Preoperative features				
Age (year)	59.00 (51.00, 68.00)	59.00 (52.00, 68.00)	0.214	0.064
Male	844 (60.3)	348 (57.9)	0.344	0.048
BMI (kg/m^2^)	24.00 (21.80, 26.60)	24.10 (21.97, 26.10)	0.506	0.036
NYHA class≥III	893 (63.8)	341 (56.7)	0.003	0.144
Smoke history	180 (12.9)	72 (12.0)	0.639	0.027
Drunk history	123 (8.8)	80 (13.3)	0.003	0.145
Hypotension	668 (47.7)	300 (49.9)	0.393	0.044
Diabetes with insulin	187 (13.4)	85 (14.1)	0.69	0.023
Stroke	103 (7.4)	17 (2.8)	<0.001	0.207
CAD	324 (23.1)	156 (26.0)	0.196	0.065
MI	33 (2.4)	24 (4.0)	0.061	0.093
eGFR<60 ml/min	35 (2.5)	16 (2.7)	0.955	0.01
COPD	7 (0.5)	6 (1.0)	0.333	0.058
Peripheral arterial disease	11 (0.8)	6 (1.0)	0.834	0.023
LVEF	55.00 (51.00, 57.00)	57.00 (52.00, 61.00)	<0.001	0.325
SOFA score≥1	617 (44.1)	250 (41.6)	0.33	0.05
Albumin (g/L)	39.80 (37.70, 41.60)	39.90 (37.70, 41.90)	0.17	0.092
History of cardiac surgery	86 (6.1)	22 (3.7)	0.032	0.115
Operative features				
Emergency surgery	161 (11.5)	29 (4.8)	<0.001	0.246
Procedure name			0.002	0.213
Isolated CABG	192 (13.7)	121 (20.1)		
AVR or MVR or TVR	525 (37.5)	235 (39.1)		
AVR + MVR	175 (12.5)	60 (10.0)		
Valve + CABG surgery	83 (5.9)	38 (6.3)		
Thoracic aortic surgery	327 (23.4)	109 (18.1)		
Others	98 (7.0)	38 (6.3)		
CPB (min)	126.00 (88.00, 170.00)	126.00 (86.00, 165.00)	0.304	0.088
ACC (min)	88.00 (57.00, 126.00)	88.00 (35.00, 120.00)	0.091	0.113
DHCA (min)	0.00 (0.00, 0.00)	0.00 (0.00, 0.00)	0.365	0.006
Minimally invasive	230 (16.4)	89 (14.8)	0.4	0.045
Transfusion				
RBCs (u)	0.50 (0.00, 4.00)	0.00 (0.00, 4.00)	0.666	0.055
Plasma (ml)	0.00 (0.00, 600.00)	0.00 (0.00, 575.00)	0.146	0.127
Platelet	323 (23.1)	124 (20.6)	0.253	0.059
Cryoprecipitate	344 (24.6)	118 (19.6)	0.019	0.119
Postoperative features				
Use of ECMO or IABP or CRRT	40 (2.9)	14 (2.3)	0.605	0.033
CXR exudation			0.139	0.095
Mild	23 (1.6)	7 (1.2)		
Moderate	1302 (93.0)	549 (91.3)		
Severe	75 (5.4)	45 (7.5)		
Duration of MV (h)	9.00 (5.00, 17.50)	10.40 (6.00, 19.00)	0.009	0.084
Procalcitonin on POD3 (ng/ml)	1.96 (0.67, 5.81)	3.20 (0.93, 13.50)	<0.001	0.340
C-reactive protein on POD3 (ng/ml)	117.02 (81.66, 161.26)	147.65 (83.00, 220.80)	<0.001	0.323
Interleukin-6 on POD3 (ng/ml)	42.13 (25.71, 87.10)	35.79 (23.27, 69.84)	<0.001	0.001

SMD: standardized mean difference; BMI: body mass index; NYHA: New York Heart Association Functional Classification; CAD: coronary artery disease; MI: myocardial infarction; LVEF: left ventricular ejection fraction; SOFA: sequential organ failure assessment; CABG: coronary artery bypass graft; AVR: aortic valve repair or replacement; MVR: mitral valve repair or replacement; TVR: tricuspid valve repair or replacement;; CPB: cardio pulmonary bypass; ACC: aortic cross clamp; DHCA: deep hypothermia circulatory arrest; CXR exudation: chest X-ray exudation; ECMO: extra corporeal membrane oxygenation; IABP: intra-aortic balloon pump; CRRT: continuous renal replacement therapy; MV: mechanical ventilation; POD: postoperative days.

### Clinical outcomes in the training and validation datasets

**Figure 2.**
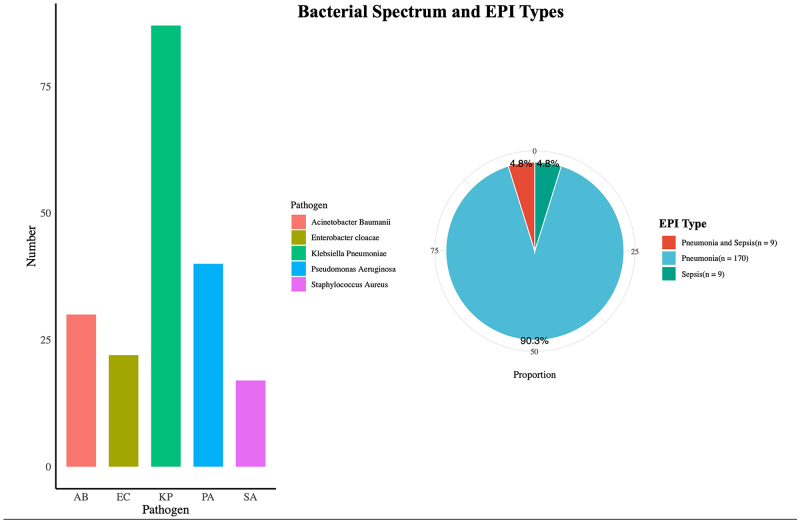
Bacterial species and type of early postoperative infections.

Out of 2001 patients, 188 (9.39%) experienced EPI, with incidences varying slightly across hospitals. Pneumonia was the most common EPI (Fig. 2), with no cases of surgical site infection or intravenous catheter-related infections. Among the EPI cases, the most frequently identified bacterial strains were *Klebsiella* (*n* = 92), *Pseudomonas aeruginosa* (*n* = 40), *Acinetobacter baumannii* (*n* = 30), *Enterobacter cloacae* (*n* = 22), and *Staphylococcus aureus* (*n* = 17). Mixed infections occurred in 14 patients. Only 42 EPI patients (22.3%) received stronger antibiotics on POD3, while the rest (*n* = 146) continued with first- or second-generation cephalosporins (Supplemental Digital Content, Fig. S2, http://links.lww.com/JS9/D861). These 146 patients were later upgraded to antibiotics such as cefoperazone-sulbactam (SCF), imipenem (IPM), and meropenem (MEM). Among the 1813 patients without EPI, 272 patients (15%) were given stronger antibiotics on POD3 (Supplemental Digital Content, Fig. S2, http://links.lww.com/JS9/D861). Table 2 provides a comprehensive overview of the clinical outcomes observed in the EPI group and the control group across the training and validation datasets. In the training dataset, patients in the EPI group exhibited significantly prolonged durations of mechanical ventilation (19.0 hours vs. 8.0 hours, *P* < 0.001), duration of intensive care unit (ICU) stay (4.0 days vs. 2.0 days, *P* < 0.001), etc. These trends were consistent in the validation dataset.

**Table 2:** Clinical outcomes of patients in the EPI and control groups

	Training dataset			Validation dataset		
	EPI (*n* = 129)	Control (*n* = 1271)	*P*- value	EPI (*n* = 59)	Control (*n* = 542)	*P*- value
Duration of MV, hours	19.0 (8.5, 72.0)	8.0 (5.0, 17.0)	<0.001	20.0 (9.3, 44.00)	10.0 (6.0, 18.0)	<0.001
Duration of ICU, days	4.0 (3.0, 10.0)	2.0 (2.0, 4.0)	<0.001	3.0 (2.0, 5.5)	3.0 (2.0, 3.0)	0.005
LOS, days	21.0 (16.0, 29.0)	17.0 (14.0, 21.0)	<0.001	23.0 (18.5, 37.0)	17.0 (15.0, 21.0)	<0.001
Death	20 (15.5)	10 (0.8)	<0.001	9 (15.3)	5 (0.9)	<0.001

EPI: early postoperative infection; MV: mechanical ventilation; ICU: intensive care unit; LOS: length of hospital stay.

### Model performances

**Figure 3.**
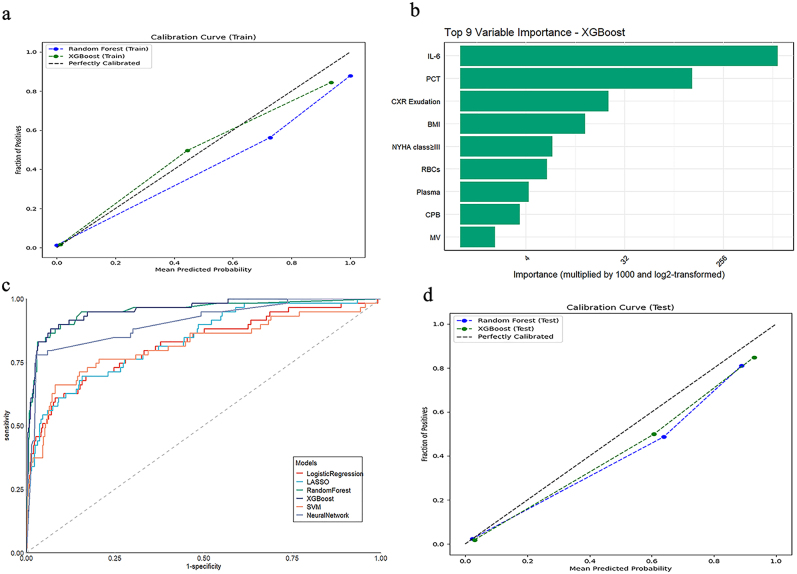
Model performance. (A) Calibration curve for XGBoost and random forest in the training set. (B) The top nine variable importance for XGBoost. (C) The AUCs of six different models. (D) Calibration curve for XGBoost and random forest in the validation set. CXR exudation: chest X-ray exudation; IL-6: interleukin-6; MV: mechanical ventilation; CPB: cardiopulmonary bypass; PCT: procalcitonin; RBC: red blood cell; NYHA class: New York Heart Association class.

In Table 3 and Figure 3c, among the six models evaluated on the external validation data, the XGBoost model exhibited the best performance with an AUROC of 0.96 (95% CI: 0.93–0.98). In comparison, LASSO achieved an AUROC of 0.84 (95% CI: 0.78–0.89), RF achieved 0.95 (95% CI: 0.92–0.99), SVM achieved 0.82 (95% CI: 0.74–0.89), LR achieved 0.82 (95% CI: 0.76–0.89), and SLNN achieved 0.91 (95% CI: 0.86–0.95). Figure 3b highlights the top nine predictors of EPI in the XGBoost model, with IL-6 on POD3 consistently emerging as the most critical factor for predicting infection. A simplified model retrained exclusively using these nine predictors achieved an AUROC of 0.94 on the validation set (Table S4 http://links.lww.com/JS9/D861).

**Table 3:** The performance of various models in diagnosing EPI on the external validation dataset

	LR	LASSO	RF	SVM	SLNN	XGBoost
AUC, (95% CI)	0.82	0.84	0.95	0.82	0.91	0.96
	(0.76–0.89)	(0.78–0.89)	(0.92–0.99)	(0.74–0.89)	(0.86–0.95)	(0.93–0.98)
Threshold = 10%						
Accuracy	0.70	0.71	0.91	0.90	0.92	0.93
Kappa	0.21	0.21	0.61	0.03	0.63	0.68
Sensitivity	0.76	0.76	0.88	0.02	0.80	0.86
Specificity	0.69	0.70	0.91	1.00	0.94	0.94
PPV	0.21	0.22	0.52	0.50	0.58	0.61
NPV	0.96	0.96	0.99	0.90	0.98	0.98
Recall	0.76	0.76	0.88	0.02	0.80	0.86
F score	0.33	0.34	0.65	0.03	0.67	0.71
Threshold = 20%						
Accuracy	0.85	0.85	0.93	0.90	0.93	0.94
Kappa	0.37	0.38	0.67	0.00	0.64	0.69
Sensitivity	0.63	0.63	0.85	0.00	0.80	0.83
Specificity	0.87	0.88	0.94	1.00	0.94	0.95
PPV	0.35	0.36	0.60	NA	0.59	0.64
NPV	0.96	0.96	0.98	0.90	0.98	0.98
Recall	0.63	0.63	0.84	0.00	0.80	0.83
F score	0.45	0.45	0.70	NA	0.68	0.73
Threshold = 50%						
Accuracy	0.92	0.92	0.95	0.90	0.93	0.95
Kappa	0.48	0.42	0.66	0.00	0.49	0.67
Sensitivity	0.42	0.36	0.59	0.00	0.41	0.59
Specificity	0.98	0.98	0.99	1.00	0.99	0.99
PPV	0.68	0.64	0.81	NA	0.75	0.83
NPV	0.94	0.93	0.96	0.90	0.94	0.96
Recall	0.42	0.36	0.59	0.00	0.41	0.59
F score	0.52	0.46	0.69	NA	0.53	0.69

EPI: early postoperative infection; SLNN: single-layer neural network; LR: logistic regression; LASSO: least absolute shrinkage and selection operator; RF: random forest; XGBoost: eXtreme gradient boosting; AUC: area under the receiver operating characteristic curve; PPV: positive predictive values; NPV: negative predictive values; NA: not available.

The calibration curve on the training datasets indicated good agreement between the predicted probabilities of XGBoost and random forest models and the observed event probabilities with Brier scores of 0.02 and 0.005, respectively (Fig. 3a). In the external validation dataset, the XGBoost model demonstrated superior performance with a cutoff of 10%, as evidenced by calibration curves and a Brier score of 0.04 (Fig. 3d). Further calibration plots stratified by age and surgical procedures showed that the XGBoost model maintained acceptable performance, as shown in Supplemental Digital Content (Fig. S5, http://links.lww.com/JS9/D861). More information on model performance, comparisons, and rationale for final model selection is available in Supplemental Digital Content.

### Model implementation

Among the matched patients, in Table 4, the DEICS group showed significantly decreased antibiotic use density (AUD) (median: 52.63 vs. 68.42, *P* < 0.001) and lower nursing-related costs (median: ¥0.29 × 10^4^ vs. ¥0.31 × 10^4^, *P* < 0.001), suggesting that DEICS may help alleviate nursing workload while also reducing antibiotic use and hospital costs. Specifically, among EPI patients, the DEICS group had a significantly shorter ICU duration compared to the control group (median: 5 days vs. 6 days, *P* = 0.014). Although the total length of stay (LOS) did not reach statistical significance, the DEICS group showed a trend toward a shorter duration (median: 21 days vs. 25.5 days, *P* = 0.052). More notably, the DEICS group demonstrated a significantly lower AUD compared to the control group (median: 60.87 vs. 75.00, *P* < 0.001), indicating more efficient antibiotic management.

**Table 4:** Outcomes in the matched-control cohort and EPI cohort

	Matched-control cohort			EPI cohort		
	DEICS (*n* = 55)	Control (*n* = 55)	*P*- value	DEICS (*n* = 29)	Control (*n* = 30)	*P*- value
EPI	29 (52.7)	30 (54.5)	1.000	NA	NA	NA
Duration of MV, hours	14.00 (6.00, 20.90)	14.00 (6.00, 23.85)	0.717	14.00 (7.00, 21.00)	16.75 (6.00, 27.00)	0.671
Duration of ICU, days	4.00 (2.50, 5.00)	5.00 (3.00, 6.00)	0.097	5.00 (4.00, 6.00)	6.00 (5.00, 7.00)	0.014
LOS, days	19.00 (17.00, 22.00)	21.00 (16.00, 26.00)	0.078	21.00 (20.00, 23.00)	25.50 (20.00, 29.00)	0.052
Death	2 (3.6)	4 (7.3)	0.675	2 (6.9)	3 (10.0)	1
AUD	52.63 (42.16, 67.95)	68.42 (50.00, 75.00)	0.008	60.87 (50.67, 71.43)	75.00 (75.00, 79.13)	<0.001
Inpatient expenses (RMB/¥, × 10^4^)	13.97 (11.93, 20.56)	14.32 (11.94, 17.46)	0.101	15.30 (10.83, 20.96)	20.05 (14.06, 28.88)	0.017
Antibiotic administration costs (RMB/¥, × 10^4^)	0.20 (0.06, 0.41)	0.23 (0.07, 0.51)	0.113	0.35 (0.12, 0.44)	0.42 (0.22, 0.58)	<0.001
Non-antibiotic pharmaceutical costs (RMB/¥, × 10^4^)	3.32 (1.88, 4.77)	3.57 (1.96, 5.41)	0.091	4.14 (1.97, 6.08)	4.71 (2.27, 7.01)	0.046
Surgical fees (RMB/¥, × 10^4^)	1.42 (0.97-1.77)	1.51 (1.08-1.89)	0.473	1.63 (1.16, 2.05)	1.65 (1.18, 2.14)	0.970
Consumable materials costs (RMB/¥, × 10^4^)	5.17 (3.07, 9.58)	5.29 (3.12, 9.71)	0.093	5.63 (3.07, 11.41)	5.78 (3.21, 11.61)	0.733
Ward and nursing care expenses (RMB/¥, × 10^4^)	0.29 (0.19, 0.50)	0.31 (0.21, 0.57)	<0.001	0.30 (0.23, 0.56)	0.41 (0.34, 0.76)	<0.001
Others (RMB/¥, × 10^4^)	3.47 (2.58, 4.62)	3.67 (2.87, 5.12)	<0.001	4.27 (2.91, 6.14)	6.14 (4.15, 9.67)	<0.001

EPI: early postoperative infection; MV: mechanical ventilation; ICU: intensive care unit; LOS: length of hospital stay; AUD: antibiotic usage density.

In terms of hospital expenses, the DEICS group had significantly lower inpatient costs (median: ¥15 300 vs. ¥20 050, *P* = 0.017), with a marked reduction in antibiotic administration costs (median: ¥0.35 × 10^4^ vs. ¥0.42 × 10^4^, *P* < 0.001). Non-antibiotic pharmaceutical costs also decreased significantly (median: ¥4.14 × 10^4^ vs. ¥4.71 × 10^4^, *P* = 0.046). Furthermore, nursing-related expenses were significantly lower in the DEICS group (median: ¥0.29 × 10^4^ vs. ¥0.31 × 10^4^, *P* < 0.001). This indicates that DEICS plays a critical role in optimizing infection management, reducing resource consumption during hospitalization, and potentially enhancing nursing efficiency.

All patients in the DEICS group upgraded antibiotics on POD3, compared to only 54.55% in the control group. The sensitivity of the XGBoost model was 100%, compared to 22.58% for clinical judgment. Both the control group and the DEICS group had similar numbers of false positives (*n* = 25 vs. 26, respectively), resulting in non-EPI patients being incorrectly administered upgraded antibiotics (Supplemental Digital Content, Table S8, http://links.lww.com/JS9/D861 and Fig. S4, http://links.lww.com/JS9/D861). However, the positive predictive value (PPV) of the XGBoost model was significantly higher than clinical judgment (52.73% vs. 22.58%, Table S8 http://links.lww.com/JS9/D861). Additional results, including antibiotic coverage accuracy, baseline characteristics between the two groups, and other outcomes, can be found in Supplemental Digital Content.

## Discussion

This prospective, multi-center modeling study evaluated the performance of various machine-learning methods in diagnosing EPI following cardiac surgery. The study found that machine learning-based models generally outperformed traditional logistic regression, achieving higher AUROCs. Among these models, the XGBoost model demonstrated the best performance in the validation set, with an AUROC of 0.96. Furthermore, in clinical application, the XGBoost model showed potential in reducing antibiotic usage density (AUD), ICU stay duration, and hospital costs, presenting a valuable alternative to traditional clinical judgment.

Recently, a multi-center study involving over 70 000 patients reinforced earlier findings identifying important perioperative risk factors for pneumonia, including cardiopulmonary bypass time greater than 90 minutes, extubating more than six hours post-operation, transfusion of blood products, and the use of an intra-aortic balloon pump. This study, utilizing logistic regression modeling, achieved a C-statistic of 0.771^[2]^, similar to previous research^[6,12,13]^. These models primarily focused on predicting the risk of broader hospital-acquired infections. However, there remains a critical gap in developing models capable of diagnosing EPI early and accurately.

In this study, we aimed to develop a diagnostic model specifically for EPI, incorporating preoperative baseline data, intraoperative, and postoperative clinical information. The primary application of this model is to identify whether a patient has developed a bacterial infection in the early postoperative period, thereby guiding clinical decisions on whether to continue antibiotic treatment. Our model bridges this gap by utilizing baseline clinical data and laboratory results collected on the morning of postoperative day 3.

The XGBoost model exhibited exceptional performance, achieving an AUROC score of 0.96 and a 93% accuracy rate, significantly outperforming previous models and demonstrating high accuracy and specificity in this critical window. This performance surpassed that of clinical judgement in guiding antibiotic upgrades for EPI patients, enabling swift and accurate adjustments to antibiotic regimen. The high sensitivity and PPV of the XGBoost model compared to clinical empirical judgement is important as missed diagnoses pose a greater risk than misdiagnoses for EPI. Most control group patients retained insufficient first- or second-generation cephalosporins, causing a delayed escalation by 2–3 days and the subsequent use of stronger antibiotics like imipenem, meropenem, and cefoperazone-sulbactam, leading to prolonged antibiotic use. In contrast, in the DEICS group, early escalated antibiotics on POD3 comprised piperacillin-tazobactam, ceftolozane-tazobactam, and cefoperazone sodium, with a shorter duration of antibiotic use. This potentially explains the decreased AUD in the DEICS group. Delayed diagnosis and treatment can intensify infections, prolong antibiotic use, and elevate the risk of antimicrobial resistance and healthcare costs^[19,20]^. Previous studies have revealed substantial cost increases associated with postoperative infections^[19,21–24]^. Our study provides direct evidence that early adoption of a machine-learning model provides real-time guidance for antibiotic management, improving patient outcomes and reducing costs.

However, it is important to note that when the diagnostic threshold is set at the optimal cutoff, 42.86% (39 out of 91 cases) of patients present false positives, while 1.4% (7 out of 510 cases) of patients exhibit false negatives. Although the high false-positive rate may lead to inappropriate antibiotic use in this subset of patients, the intensity and cost of antibiotic use in the model group are significantly lower compared to reliance solely on clinical experience without the model.

We observed that as the diagnostic threshold increases, the likelihood of false positives significantly decreases; however, the proportion of false negatives correspondingly increases. When the infection risk threshold is set at 50%, the false-positive rate drops significantly to 16.7% (7 out of 42 cases), but at the same time, the false-negative rate increases three-fold, rising to 4.3% (24 out of 559 cases) (Supplemental Digital Content, Fig. S3, http://links.lww.com/JS9/D861).

Considering the catastrophic consequences of bacterial infections following cardiac surgery, clinical experts recommend a more lenient tolerance for false positives to minimize the possibility of false negatives. Consequently, we ultimately suggest using an infection risk greater than 10% as the threshold for guiding the continuation of antibiotics. At this threshold, the false-positive rate is 39.29% (33 out of 84 cases), while the false-negative rate is 1.5% (8 out of 517 cases).

Embedding the model into electronic medical record (EMR) systems could significantly enhance its clinical utility by facilitating automated data extraction and risk calculation. This integration would reduce clinicians’ workload and promote the model’s routine use in clinical practice. However, for economically underdeveloped regions, using a simplified version of the XGBoost model may be a more suitable option. The model’s inherent complexity, driven by its integration of advanced features, could present challenges to widespread adoption in such areas. To address these challenges, we are exploring ways to simplify the model further while retaining its diagnostic accuracy.

We aspire to conduct a multinational, multi-center randomized controlled trial in the near future to evaluate the diagnostic value and generalizability of our model. We look forward to the model being applied across different centers, allowing for its evaluation of the diagnostic value and generalizability in diverse patient populations. Last, we will continue to iterate and improve the model to enhance its simplicity and usability.

In conclusion, we have developed an accurate and robust machine-learning model for diagnosis of EPI after cardiac surgery. Its precise and timely recommendations for antibiotic upgrades present a substantial improvement over current clinical practices, highlighting its value as a critical tool in postoperative care. We advocate for its early adoption on the morning of POD3 to improve clinical outcomes and resource efficiency in cardiac surgery.

### Limitation

Our study has several limitations. First, the model does not account for varying levels of infection severity, which may limit its clinical applicability, particularly in distinguishing between mild and severe infections. Second, there is potential selection bias due to the inclusion of emergency surgery cases, which may not fully reflect the broader patient population and could impact the generalizability of the results. Third, while we evaluated five machine-learning models, there is potential for further improvement by exploring newer or more advanced algorithms in future work. Fourth, the relatively small sample size constrained our ability to perform a propensity-matched analysis, which could have helped minimize bias in treatment allocation.

Additionally, the study was conducted in a single country, which may limit the generalizability of the model’s diagnostic performance to other healthcare systems with different clinical practices and insurance structures. Most notably, the lack of antimicrobial resistance (AMR) data is a critical gap in our current model. AMR plays a significant role in clinical decision-making and the effectiveness of antibiotic therapies, and its omission may limit the model’s practical utility. We recognize the importance of incorporating AMR data into future studies and will prioritize the collection of relevant antimicrobial resistance patterns to enhance the model’s accuracy and clinical relevance. We plan to explicitly address this limitation in the discussion section and propose the inclusion of AMR data as a key objective in subsequent research initiatives.

To address these limitations, we propose conducting a randomized controlled trial to further evaluate the XGBoost model’s performance and explore its broader applicability across diverse populations and healthcare systems.

## Data Availability

The data utilized in this study can be made available via the following Web address: https://pan.baidu.com/s/1TYGE8JxzCKRp12ebdf7MFg.

## Research registration unique identifying number (UIN)

The trial was registered at the Chinese Clinical Trial Register (www.chictr.org.cn, No: ChiCTR2000038762 and ChiCTR2200058193).

## Web calculator

https://dicsmodels.shinyapps.io/DEICS_calculator/ and https://dicsmodels.shinyapps.io/Simplified-DEICS/.

## References

[1] Greco G Shi W Michler RE. Costs associated with health care-associated infections in cardiac surgery. J Am Coll Cardiol 2015;65:15–23. 25572505 10.1016/j.jacc.2014.09.079 PMC4293042

[2] Barnett NM Liesman DR Strobel RJ. The association of intraoperative and early postoperative events with risk of pneumonia following cardiac surgery. J Thorac Cardiovasc Surg 2024;168:1144–54e3. 37797934 10.1016/j.jtcvs.2023.09.056 PMC10991082

[3] Jiang WL Hu XP Hu ZP. Morbidity and mortality of nosocomial infection after cardiovascular surgery: a report of 1606 cases. Curr Med Sci 2018;38:329–35. 30074193 10.1007/s11596-018-1883-4

[4] He S Chen B Li W. Ventilator-associated pneumonia after cardiac surgery: a meta-analysis and systematic review. J Thorac Cardiovasc Surg 2014;148:3148–55e1–5. 25240522 10.1016/j.jtcvs.2014.07.107

[5] Brown PP Kugelmass AD Cohen DJ. The frequency and cost of complications associated with coronary artery bypass grafting surgery: results from the United States medicare program. Ann Thorac Surg 2008;85:1980–86. 18498806 10.1016/j.athoracsur.2008.01.053

[6] Zhang HT Wang K Li ZS. Diagnosis of early bacterial pneumonia and sepsis after cardiovascular surgery: a diagnostic prediction model based on LASSO logistic regression. J Inflamm Res 2023;16:3983–96. 37719939 10.2147/JIR.S423683 PMC10503509

[7] Li Q Zheng S Zhou PY. The diagnostic accuracy of procalcitonin in infectious patients after cardiac surgery: a systematic review and meta-analysis. J Cardiovasc Med (Hagerstown) 2021;22:305–12. 33633046 10.2459/JCM.0000000000001017

[8] Bratzler DW Dellinger EP Olsen KM. Clinical practice guidelines for antimicrobial prophylaxis in surgery. Am J Health Syst Pharm 2013;70:195–283. 23327981 10.2146/ajhp120568

[9] Berrios-Torres SI Umscheid CA Bratzler DW. Centers for disease control and prevention guideline for the prevention of surgical site infection, 2017. JAMA Surg 2017;152:784–91. 28467526 10.1001/jamasurg.2017.0904

[10] Alleyne CH Jr Hassan M Zabramski JM. The efficacy and cost of prophylactic and perioprocedural antibiotics in patients with external ventricular drains. Neurosurgery 2000;47:1124–27. 11063105 10.1097/00006123-200011000-00020

[11] Kilic A Ohkuma R Grimm JC. A novel score to estimate the risk of pneumonia after cardiac surgery. J Thorac Cardiovasc Surg 2016;151:1415–20. 27085620 10.1016/j.jtcvs.2015.12.049

[12] Wang D Li Y Sheng W. Development and validation of a nomogram model for pneumonia after redo cardiac surgery. J Cardiovasc Med (Hagerstown) 2022;23:325–34. 37594436 10.2459/JCM.0000000000001302

[13] Gao Y Wang C Wang Y. Establishment and validation of a nomogram to predict hospital-acquired infection in elderly patients after cardiac surgery. Clin Interv Aging 2022;17:141–50. 35173428 10.2147/CIA.S351226 PMC8841270

[14] Li B Verma R Beaton D. Predicting major adverse cardiovascular events following carotid endarterectomy using machine learning. J Am Heart Assoc 2023;12:e030508. 37804197 10.1161/JAHA.123.030508 PMC10757546

[15] Collins GS Reitsma JB Altman DG. Transparent reporting of a multivariable prediction model for individual prognosis or diagnosis (TRIPOD): the TRIPOD statement. BMJ 2015;350:g7594. 25569120 10.1136/bmj.g7594

[16] Mathew G Agha R Albrecht J. STROCSS 2021: strengthening the reporting of cohort, cross-sectional and case-control studies in surgery. Int J Surg 2021;96:106165. 34774726 10.1016/j.ijsu.2021.106165

[17] Zhang HT Han XK Wang CS. Diagnosis of infection after cardiovascular surgery (DICS): a study protocol for developing and validating a prediction model in prospective observational study. BMJ Open 2021;11:e048310. 10.1136/bmjopen-2020-048310 PMC8458369 34548352

[18] Chen W Zhong K Guan Y. Evaluation of the significance of interleukin-6 in the diagnosis of postoperative pneumonia: a prospective study. BMC Cardiovasc Disord 2022;22:306. 35794529 10.1186/s12872-022-02744-0 PMC9261039

[19] Ailawadi G Chang HL O’Gara PT. Pneumonia after cardiac surgery: experience of the national institutes of health/canadian institutes of health research cardiothoracic surgical trials network. J Thorac Cardiovasc Surg 2017;153:1384–91e3. 28341473 10.1016/j.jtcvs.2016.12.055 PMC5439299

[20] Hadaya J Downey P Tran Z. Impact of postoperative infections on readmission and resource use in elective cardiac surgery. Ann Thorac Surg 2022;113:774–82. 33882295 10.1016/j.athoracsur.2021.04.013

[21] Iribarne A Burgener JD Hong K. Quantifying the incremental cost of complications associated with mitral valve surgery in the United States. J Thorac Cardiovasc Surg 2012;143:864–72. 22424521 10.1016/j.jtcvs.2012.01.032

[22] Kelava M Robich M Houghtaling PL. Hospitalization before surgery increases risk for postoperative infections. J Thorac Cardiovasc Surg 2014;148:1615–21e3. 25260276 10.1016/j.jtcvs.2014.06.067

[23] Mehaffey JH Hawkins RB Byler M. Cost of individual complications following coronary artery bypass grafting. J Thorac Cardiovasc Surg 2018;155:875–82e1. 29248284 10.1016/j.jtcvs.2017.08.144

[24] Thompson MP Cabrera L Strobel RJ. Association between postoperative pneumonia and 90-day episode payments and outcomes among medicare beneficiaries undergoing cardiac surgery. Circ Cardiovasc Qual Outcomes 2018;11:e004818. 30354549 10.1161/CIRCOUTCOMES.118.004818

